# Unravelling the Water Adsorption Mechanism in Hierarchical MOFs: Insights from In Situ Positron Annihilation Lifetime Studies

**DOI:** 10.1021/acsami.3c10974

**Published:** 2023-10-05

**Authors:** Ahmed G. Attallah, Volodymyr Bon, Kartik Maity, Eric Hirschmann, Maik Butterling, Andreas Wagner, Stefan Kaskel

**Affiliations:** † Helmholtz-Zentrum Dresden-Rossendorf, Institute of Radiation Physics, Dresden 01328, Germany; ‡ Physics Department, Faculty of Science, Minia University, Minia 61519, Egypt; § Chair of Inorganic Chemistry I, Technische Universität Dresden, Bergstrasse 66, Dresden D-01062, Germany; ∥ Fraunhofer Institute for Material and Beam Technology IWS, Winterbergstraße 28, Dresden D01277, Germany

**Keywords:** MOFs, DUT-67, positron annihilation lifetime spectroscopy, sorption mechanism, water harvesting

## Abstract

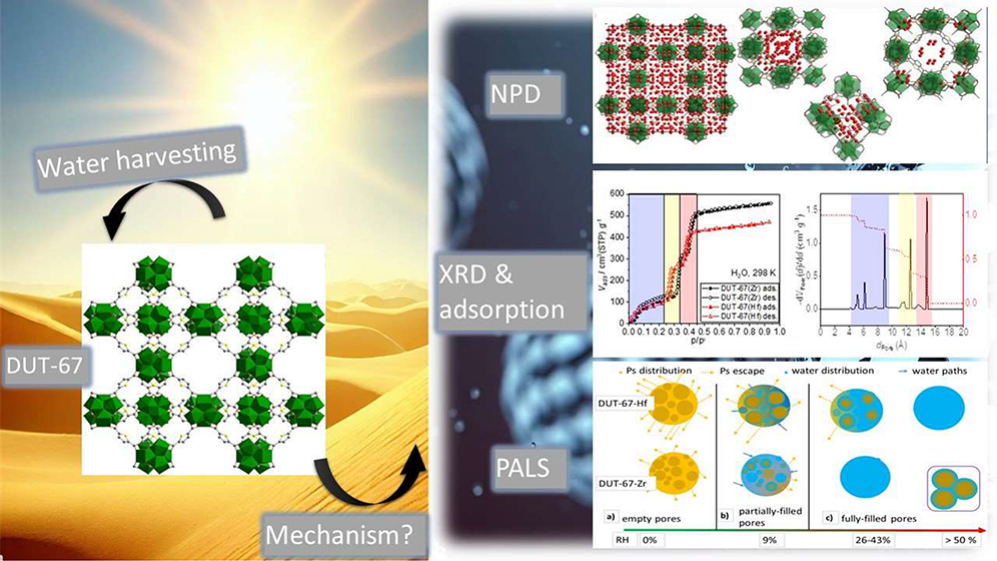

Atmospheric water harvesting with metal–organic frameworks (MOFs) is a new technology providing a clean, long-term water supply in arid areas. In-situ positron annihilation lifetime spectroscopy (PALS) is proposed as a valid methodology for the mechanistic understanding of water sorption in MOFs and the selection of prospective candidates for desired applications. DUT-67-Zr and DUT-67-Hf frameworks are used as model systems for method validation because of their hierarchical pore structure, high adsorption capacity, and chemical stability. Both frameworks are characterized using complementary techniques, such as nitrogen (77 K) and water vapor (298 K) physisorption, SEM, and PXRD. DUT-67-Zr and DUT-67-Hf are investigated by PALS upon exposure to humidity for the first time, demonstrating the stepwise pore filling mechanism by water molecules for both MOFs. In addition to exploring the potential of PALS as a tool for probing MOFs during in situ water loading, this work offers perspectives on the design and use of MOFs for water harvesting.

## Introduction

The scarcity of drinking water in many regions of the world is one of the major challenges facing the global society. Especially in regions with low relative humidity and low annual precipitation level, the problem of drinking water is particularly acute.^1^ To address this issue, one promising solution involves harvesting water from the atmosphere, where even the driest areas can contain up to 20–30% relative humidity.^2−6^ Porosity plays a significant role in water harvesting; however, a critical aspect of optimizing this process and minimizing energy consumption lies in the selection of the appropriate porous materials.^7^ Established porous solids, such as zeolites and porous carbons, are often suitable only to a limited extent since either high-energy input for desorbing the water is required or hydrophobicity causes the low adsorption capacity. In this context, the utilization of porous crystalline frameworks, such as MOFs (metal–organic frameworks) and COFs (covalent organic frameworks), offers the advantage of tuning pore characteristics to achieve optimal water adsorption at desired humidity conditions.^6^ Recently, Yaghi and co-workers developed pilot plants for water capture in desert conditions, using chemically stable MOFs as working solids, underscoring the significance of chemical stability alongside performance considerations.^8,9^

Zr-based frameworks stand out as strong contenders for water adsorption. Their unique attributes make them potential materials for efficient water harvesting. These MOFs offer diverse topologies, modulator-driven connectivity of a Zr_6_O_4_(OH)_4_^12+^ oxo-cluster, and inherent functionalization potential.^10,11^ In general, increasing the concentration of the modulator in the synthesis leads to capping of anchoring sites and systematic connectivity reduction from 12- to 4-connected frameworks.^12−14^ The use of tetratopic ligands such as porphyrin tetracarboxylate or pyrene tetracarboxylates results in the formation of stable, highly porous, and functional frameworks such as MOF-525,^15^ PCN-222,^16^ PCN-224,^17^ NU-1000,^18^ etc.

In our group we developed a series of Zr- and Hf-based MOFs, based on above mentioned Zr-cluster and the bent ligand 2,5-thiophenedicarboxylic acid, known as DUT-67, DUT-68, and DUT-69^19^ (DUT: Dresden University of Technology). These MOFs show different crystal structures, framework topologies, pore system, and high chemical stability. Water physisorption experiments emphasize DUT-67-Zr among these three solids because of the well-defined three steps in water vapor adsorption isotherm showing high adsorption capacity and reversibility. In addition, the report of green synthesis of DUT-67-Zr^20^ makes this solid affordable and highly scalable and, therefore, prospective for water harvesting applications.

It is worth noting that chemical stability is not the only prerequisite for the design of water harvesters. An essential foundation for advancing high-performance materials in this area is a thorough understanding of the mechanisms underlying water adsorption.^21−24^ In this regard, the integration of X-ray and neutron diffraction techniques, conducted in situ or on preloaded samples, has proven pivotal in exploring adsorption sites within the nanopores of MOFs.^25−28^ Neutron powder diffraction (NPD) studies on the D_2_O preloaded samples shed light on the water adsorption mechanism in these materials.^26^ For instance, NPD identified the positions of water molecules during water vapor physisorption on DUT-67-Zr and DUT-67-Hf of an S-shaped isotherm with three distinct steps.^29^

Nevertheless, PXRD and NPD techniques are quite sensitive at low loadings and can idenity strongly interacting water molecules. At higher loadings, on the other hand, the precise localization of the adsorption sites became challenging because of the high symmetry of the framework and dynamic disorder of weakly bonded water molecules in the pores. To obtain more precise information about the adsorption mechanism at higher loadings, other techniques should be applied. For instance, complementary analysis of the structure at high water loading can be provided from in situ positron annihilation lifetime spectroscopy (PALS) measurements.

PALS has been used to identify the porosity in many micro- and mesoporous structures, e.g., zeolites,^30^ MOFs,^31−33^ polymers,^34^ and porous glasses.^35^ Despite being a relatively new addition to the MOFs field,^36−38^ PALS has quickly been established as a powerful nondestructive tool for in situ analysis. Its capabilities on MOFs include probing isolated pores^31,39^ and tracking structural changes during thermal treatments,^38^ as well as providing porosity information during gas adsorption studies.^37,38^ The physical basis of PALS porosimetry relies on the principle that the annihilation lifetime of positronium (Ps), the bound state of an electron and a positron e^+^ (the antiparticle of an electron), in free volumes, is a function of the size of such voids.^40−45^ In a porous medium, Ps is formed upon exposure of the porous solid to energetic positrons that diffuse deeply into the sample. Consequently, Ps is formed by the interactions between the implanted positrons and materials’ electrons, allowing the detection of even restricted and isolated pores. The Tao-Eldrup (TE) model,^40,41^ for micropores, and its extensions,^42,46^ for mesopores, correlate the measured Ps lifetime and the pore size in monotonous dependence. The Supporting Information, section S1, provides more details about the PALS method.^47^

In chemically stable MOFs, such as the DUT-67 series, the accessibility of the pore system during water loading can be attributed solely to the progressive filling of pores without any pore collapse. This can be observed in PALS from the reduction in both Ps lifetime and intensity, indicating a decrease in the pore size and concentration, respectively. Therefore, PALS is well-suited to monitor the variation of the porosity of DUT-67 during water exposure. This motivates us to develop an *in situ* PALS methodology for monitoring the water physisorption on MOFs using DUT-67 frameworks as model systems. In the following, we employ *in situ* PALS^48,49^ for exploring the water harvesting in DUT-67-Zr and -Hf frameworks, as a proof of principle example and validation of in situ PALS methodology. The study opens new horizons toward the rational design of MOFs for impactful applications in adsorption-driven heat pumps or direct water harvesting from the air.

## Experimental section

### Material synthesis

Precursors of DUT-67-Zr and DUT-67-Hf contain 2,5-thiophenedicarboxylic acid (5.165 g, 30 mmol) and (*i*) ZrOCl_2_·8H_2_O and (*ii*) HfCl_4_ (14.501 g, 45 mmol), respectively. These precursors were added into a mixture of 90 mL of deionized water and 90 mL of glacial acetic acid while stirring in a round-bottom flask. The reaction mixture was refluxed at 95 °C for 1 h. The resulting white precipitate was collected by centrifugation and washed twice with 0.1 M sodium acetate solution and water to deprotonate and wash out the excess of ligand. After that, the material was activated at 368 K under dynamic vacuum for 15 h. The yields were 8.5 g for DUT-67-Zr and 8.2 g for DUT-67-Hf.

### Methods

*Powder X-ray diffraction (PXRD)* patterns were collected at room temperature using a STOE STADI P diffractometer equipped with a MYTHEN 100k (DECTRIS) 1D detector and X-ray tube, operated at 40 kV and 30 mA and curved Ge(111)-monochromator producing Cu Kα1 (λ = 0.15405 nm) radiation. The measurements were conducted using step scans with *Δθ* = 6° in transmission mode (*ω-2θ* scan) using a flat-bed sample holder.

*Thermogravimetric (TG)* analysis was accomplished with synthetic air flow (5 mL/min) in a temperature range of 20–1000 °C with a heating rate of 2 K/min using STA 409 PC (NETZSCH).

SEM images were recorded with secondary electrons in a SU8020 (Hitachi) operated at an acceleration voltage of 1 kV and a 10.8 mm working distance.

*Nitrogen volumetric physisorption* isotherms were measured at 77 K and performed on a Quadrasorb apparatus from Quantachrome.

*Water vapor adsorption–desorption* isotherms were measured at 298 K using BELSORP-max volumetric adsorption device.

**Figure 1:**
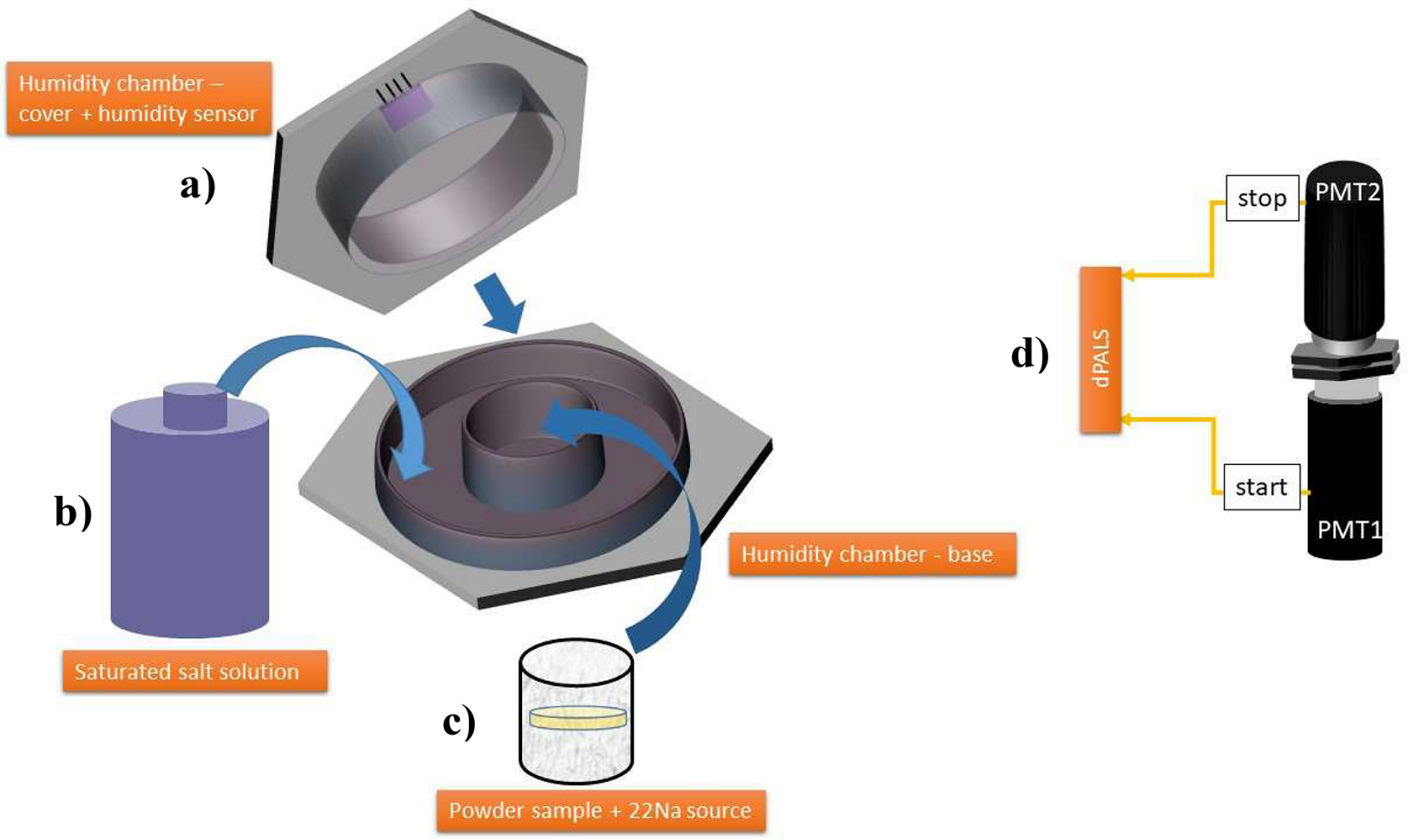
Setup for the in situ humidity variation during PALS measurements. The humidity chamber features a humidity sensor (a) with saturated salt solution (b) surrounding the Al container for the powder samples and positron source (c) was sandwiched between two photomultiplier tubes (PMTs) providing start and stop signals for digital PALS (dPALS) measurement (d).

*PALS* measurements were conducted on DUT-67 MOFs during in situ humidity exposure. Figure 1 provides an overview of the integrated humidity cell to the PALS experiment allowing *in situ* humidity control using a custom-made humidity chamber. The humidity chamber is made of PEEK and is designed in such a way that the sample, protected from the environment, is exclusively connected to the atmosphere of humid air in the chamber (Figure 1a). The chamber’s outer ring is filled with saturated salt solutions (Figure 1b) leading to well-defined adjustable RH levels (Table S1). For 100% RH, we used water instead of saturated salt solutions. By tightening of the upper part of the humidity chamber, the humidity generated by the saturated salt solution (or water) is equilibrated and allowed to intrude into the sample area. To ensure accurate measurements, a humidity sensor (DHT22) is used to monitor the humidity above the sample (Figure 1a). The sensor has a resolution of 0.1%RH and an accuracy tolerance of 3%. The humidity is monitored every 5 s to ensure that the actual humidity level is consistent with the desired level. Examples of the registered humidity during humidity exposure prior to and during PALS measurements are shown in Figure S1 and the dependence of Ps lifetime and intensity on humidity over time of DUT-67-Zr at 9% RH is shown in Figure S2. A cylindrical aluminum container of Ø = 12 mm and h = 8 mm was filled with the powder MOFs samples (∼900 mm^3^) and ^22^Na positron source with ∼630 kBq activity in the center of the powder (yellow disc in Figure 1c). The powder samples have been gently pressed to fill any possible gaps between the fine particles.

PALS measurements were conducted using a digitizer (Teledyne SP-Devices, ADQ14DC-2X-MTCA) with two photomultiplier tubes (Ortec, Model 265) coupled to two plastic scintillators (Ø = 40 mm by h = 20 mm) (Figure 1d) for the coincident detection of annihilation radiation and a customized data acquisition software.^50^ Before humidity measurements, a series of reference samples (Al, YSZ, and Ta) were used to determine source contributions (positrons not annihilating inside the sample) and time-resolution functions. The timing resolution was 240 ps at the full width of half-maximum, and ∼11% source contribution, shared between Kapton (0.38 ns, ∼ 10.8%) and glue (∼3 ns, ∼ 0.2%), was accounted for during the lifetime analysis.

Each sample was exposed to humidity for approximately 15 h, including 12 h to allow the humidity to reach equilibrium within the sample. Following that, PALS measurements commenced, with each measurement lasting approximately 3 h to collect 2 × 10^6^ events.

The analysis of the PALS spectra was done by fitting exponential decay curves at the recorded time differences histograms using the common PALSFit routine.^51^ The analysis revealed the presence of three or five components, depending on the humidity level. Each component is characterized by a lifetime (τ_n_) and an intensity (I_n_), reflecting the size and abundance of a certain annihilation site, respectively. The origins of these components are mainly categorized based on their lifetime values. Discrete analysis by PALSFit shows that the lifetimes at different RH varied in the following ranges: τ_1_= 125–250 ps, τ_2_ = 350–500 ps, τ_3_ = 1.5–4.0 ns, τ_4_ = 10–17 ns, and τ_5_ ≈ 75 ns. Therefore, these components can be identified as annihilation of (i) para-positronium (*p*-Ps–see sec. S.1 for more details) and unbound e^+^ between the chains in the organic linkers (τ_1_), (ii) unbound e^+^ inside the pores (τ_2_), and (iii-iv) ortho-positronium (*o*-Ps) in micro- and mesopores (τ_3–5_). Our focus solely lies on components related to voids and pores (*o*-Ps: τ_3–5_). The term “Ps” in the following discussion specifically refers to *o*-Ps.

## Results and discussions

### Structural information

**Figure 2:**
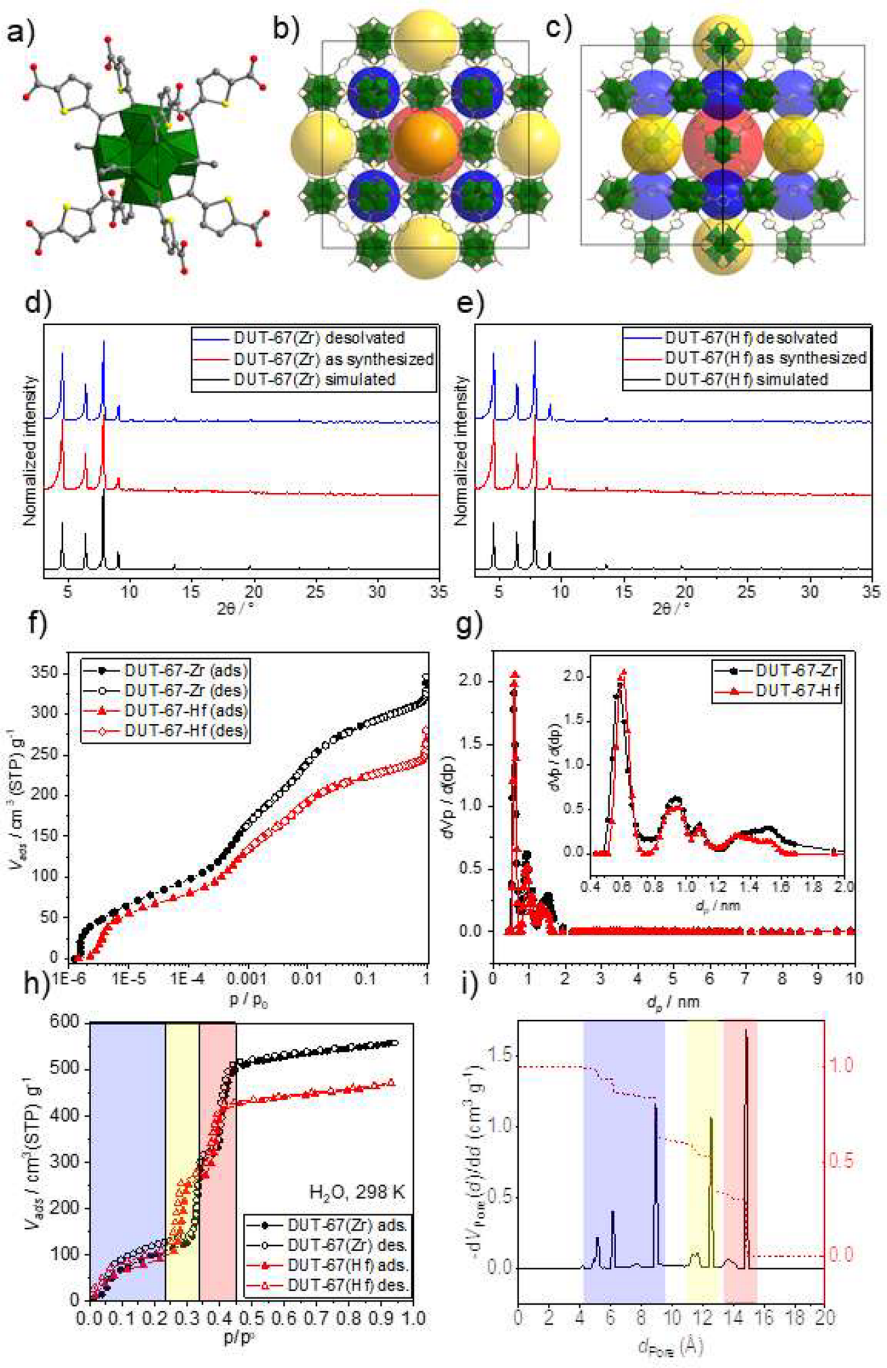
Crystal structure and characterization of DUT-67-Zr and DUT-67-Hf frameworks: (a) Inorganic node M_6_O_4_(OH)_4_^12+^ showing tdc^2–^ linkers and coordinated acetic acid; crystal structure of DUT-67 along (100) (b) and (110) (c) directions showing three different pores as blue, yellow, and red spheres; Simulated theoretical and measured as-synthesized and desolvated PXRD on DUT-67-Zr (d) and DUT-67-Hf (e); nitrogen physisorption isotherms on DUT-67-Zr and DUT-67-Hf at 77 K (f) pore size distribution for DUT-67-Zr and DUT-67-Hf, calculated from nitrogen physisorption isotherms (g); water physisorption at 298 K on DUT-67-Zr and DUT-67-Hf (h); Geometrical pore size distribution, calculated using Zeo++ software (i) showing different pores, which are present in the structure.

DUT-67-Zr and DUT-67-Hf frameworks with a composition of M_6_O_4_(OH)_4_(C_6_H_2_O_4_S)_4_(CH_3_COO)_4_ (M – Zr for DUT-67-Zr, Hf for DUT-67-Hf) are based on the corresponding M_6_O_4_(OH)_4_^12+^ inorganic nodes, each interconnected by eight 2,5-thiophenedicarboxylate linkers resulting in the 3D framework possessing **reo** topology (Figure 2a–c).^52^ If the green synthesis procedure is used, the remaining coordination sites of the metal cluster are occupied by four symmetrically coordinated acetates.^53^ Interestingly, the use of different synthesis procedures involving DMF or postsynthetic treatment of the solid may lead to a different variation of coordinated DMF/monocarboxylic acids.^54^

The synthesis procedure is upscalable to a multigram scale and produces MOFs with low defect concentration, well-defined composition of the metal cluster, and narrow distribution of crystallite size in the sample. Along with PXRD, measured after filtration and washing procedures, pure phases were obtained in both cases (Figure 2d and e). The desolvation of the MOFs at 95 °C for 15 h resulted in white crystalline powder, and no phase transitions were observed upon desolvation. SEM images show the cuboctahedral crystals with an average size of 0.4 ± 0.3 μm, which is in line with earlier reports (Figure S7c and d). The porosity of the MOFs was confirmed by nitrogen physisorption experiments showing type Ia isotherms (Figure 2f and Figures S3 and S4) and pore volumes in saturation of 0.57 and 0.47 cm^3^ g^–1^ correspondingly. These values are close to geometrical pore volume (0.60 cm^3^ g^–1^ for DUT-67-Zr and 0.45 cm^3^ g^–1^ for DUT-67-Hf) indirectly indicating the low concentration of the defects in the crystalline solids.^26^ Pore size distribution was calculated from low-pressure nitrogen physisorption experiments using GCMC methodology, implemented in BEL-Master v.7.0.18.7 (kernel for metal oxides with cylindrical pores) and shows peaks at 5.9 Å, 9.3 Å, and 10.8 Å and a broad peak in the range between 12 Å and 16 Å (Figure 2g). These numbers are well matched with theoretical values calculated from the crystal structure (Figure 2i), namely, 9 Å for the smallest octahedral cage and 12.5 Å and 14.8 Å for the middle and large cuboctahedral cages correspondingly (Figure 2i). The peaks in the pore size distribution plot at 4 Å and 6 Å correspond to the pore window located between the above-mentioned cuboctahedral cages.

Water vapor physisorption recorded at 298 K on DUT-67-Zr and DUT-67-Hf shows S-shaped isotherms with 3 steps, reflecting water adsorption in the three different pores (Figure 2h). Interestingly, water physisorption isotherms are similar to isotherms previously reported for DUT-67(Zr)-FA samples (FA, formic acid), synthesized from DMF and possessing different ratio of solvent/carboxylate, coordinated to the inorganic building unit.^29^ This indicates the major influence of the synthesis procedure on the composition and properties of Zr-MOFs with reduced framework connectivity. Along with NPD studies of the D_2_O-preloaded samples, synthesized in a water/acetic acid mixture, the pore filling starts in octahedral cages, which can be considered as the smallest pores of this structure, followed by the filling of the pores with increasing pore diameters. This filling sequence is confirmed by the steps in the water vapor isotherms.

In order to check the thermal stability, both frameworks were subjected to thermogravimetric analysis (TGA) (Figures S5 and S6). TG curves of both DUT-67-Zr and DUT-67-Hf show two steps. The first step at 110 °C is associated with the desorption of the water molecules from the pores, and the second step at ca. 425 °C corresponds to the decomposition of the MOFs.

### PALS Porosimetry

**Figure 3:**
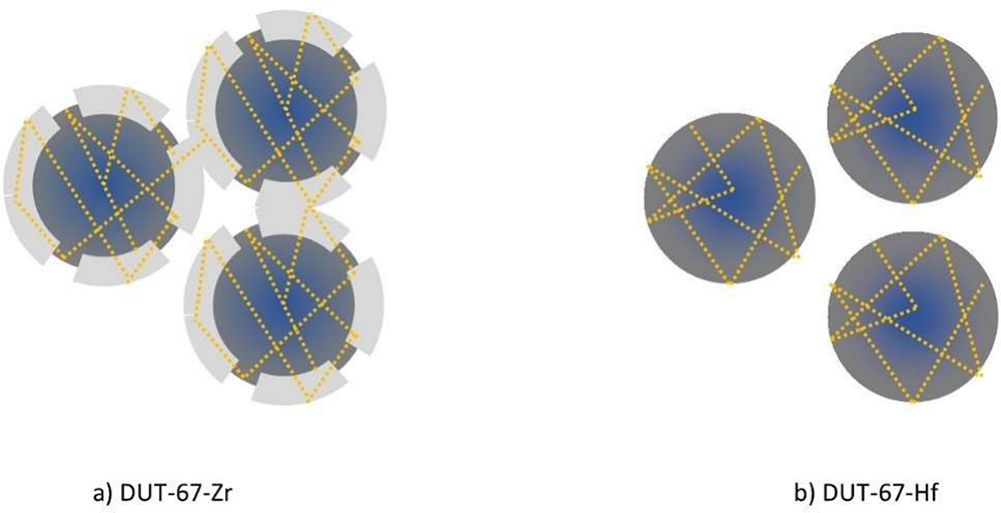
Visualization of Ps paths (dotted orange lines) inside crystals of (a) DUT-67-Zr (rough surfaces) and (b) DUT-67-Hf (flat surfaces). The rough regions in DUT-67-Zr act as bridges for Ps migration between the crystals.

**Figure 4:**
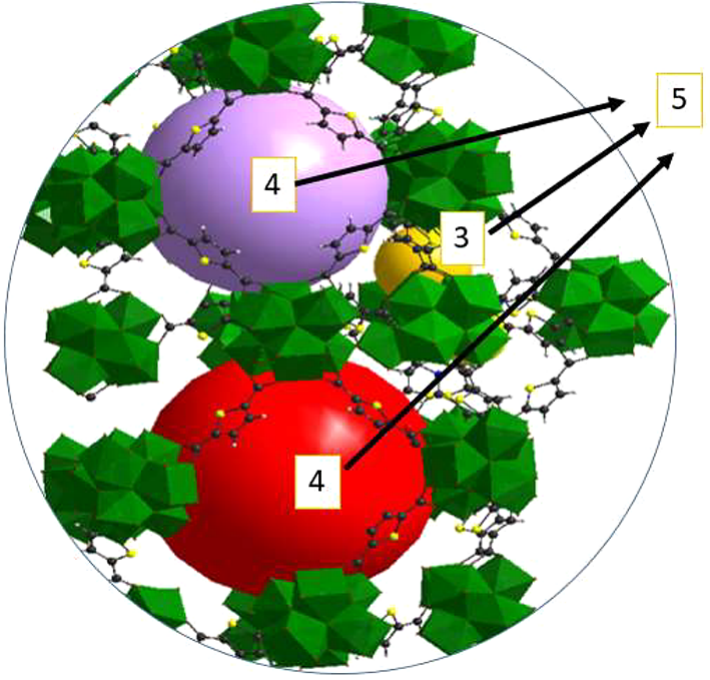
Sketch showing the possible places of Ps annihilation in DUT-67 based on the measured pore sizes by PALS compared with those calculated from crystallography. τ_3_ represents Ps annihilation in octahedral cages (yellow sphere), τ_4_ denotes Ps annihilation in middle and large cuboctahedral cages (purple and red spheres), and τ_5_ originates from Ps migration from the inner porosity to interparticle spaces.

**Figure 5:**
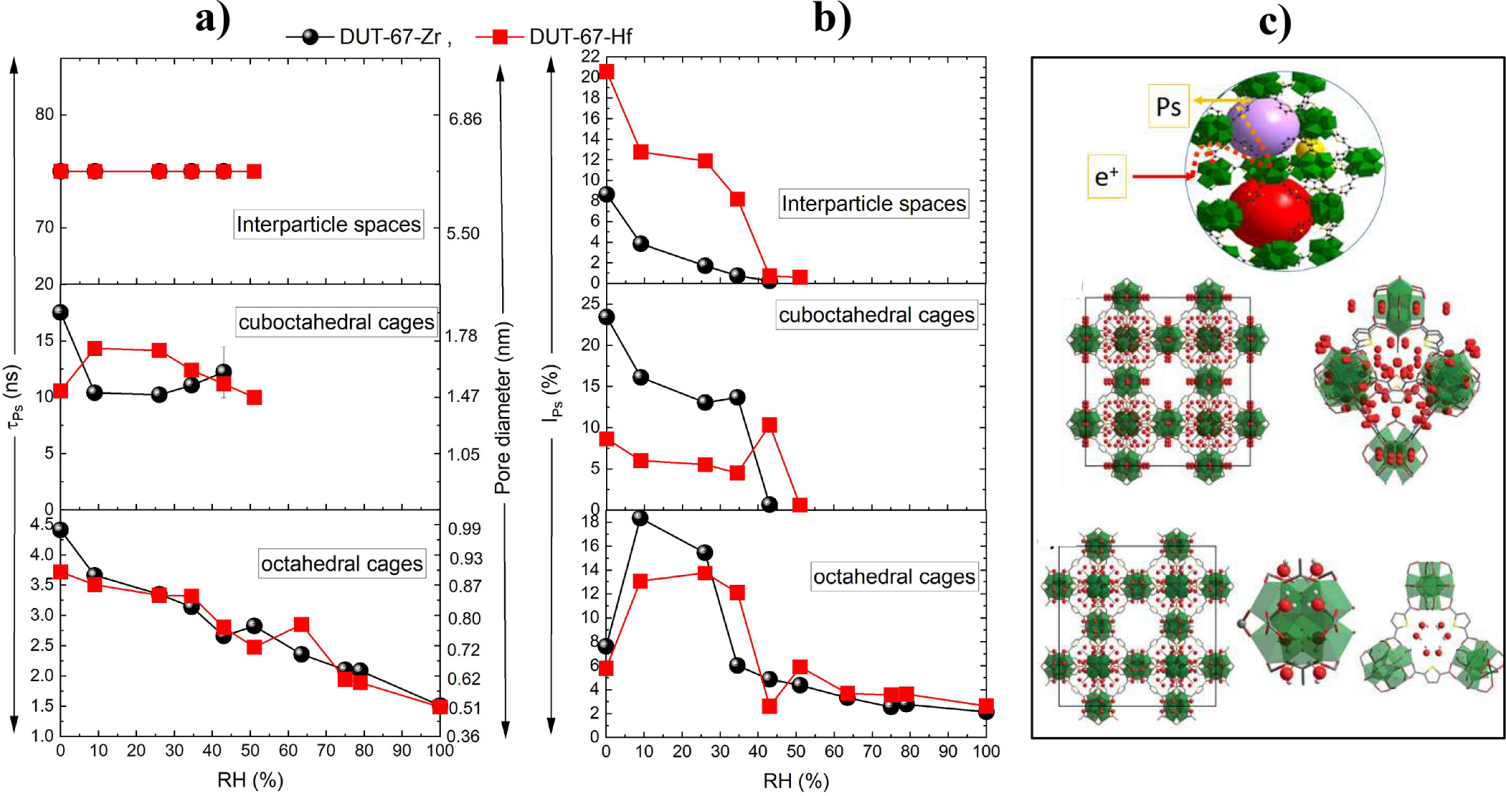
(a) Ps lifetimes (τ_Ps_) and (b) their corresponding intensities (*I*_Ps_) in octahedral cages, cuboctahedral cages, and interparticle spaces of DUT-67-Zr (black circles) and -Hf (red squares) as functions of relative humidity (error bars have the same sizes as data points). Calculated spherical pore diameters are presented in the right axis of a. (c) Crystal structures of D_2_O-loaded DUT-67 when loading with Zr cluster and octahedral cages (bottom), Zr cluster and the octahedral pore (middle), and Ps formation inside the inner porosity and escape to interparticle spaces (top).

The *dry samples* have three distinct pore-related lifetime components (τ_3–5_) in nanoseconds, along with their corresponding intensities (I_3–5_). The intensity of each lifetime component reflects the occupancy of Ps in a specific pore group. Based on the calculated pore sizes, we determined that Ps annihilates in three different types of pores. In order to assign the three Ps lifetimes to their respective pore origins in DUT-67-Zr and -Hf, we used the extended TE model to calculate the pore sizes of dry samples (under vacuum and 0% RH). Figure 3 shows a sketch of the possible paths for Ps in the samples, and Figure 4 suggests Ps formation and migration out of the crystals (further details are below). The calculated pore sizes are shown in Figure 5a and are compared with the pore sizes predicted from crystallography in Table 1. This step is important to justify the discussion about changes during the course of humidity exposure.

**Table 1:** Comparison between Sizes (nm) of Different Pores in DUT-67-Zr and -Hf As Calculated from PALS Experiments and Crystal Structures^a^

	Pore size (nm)			
	DUT-67-Zr		DUT-67-Hf	
	XRD	PALS (τ_n_)	XRD	PALS (τ_n_)
octahedral cages	0.895 ± 0.015	0.99 ± 0.01 (τ_3_)	0.895 ± 0.015	0.90 ± 0.01 (τ_3_)
middle cuboctahedral cages	1.255 ± 0.015	1.80 ± 0.01 (τ_4_)	1.255 ± 0.015	1.50 ± 0.01 (τ_4_)
large cuboctahedral cages	1.485 ± 0.015		1.485 ± 0.015	
mesopores	–	6.00 (fixed τ_5_)	–	6.00 (fixed τ_5_)

a van der Waals radii of atoms are considered. The corresponding τ_n_ from Figure 5.a of each pore size is shown in parentheses.

The comparison in Table 1 reveals that the obtained pore sizes from τ_3_ fit to octahedral cages, while those obtained from τ_4_ indicate an average Ps annihilation between middle and large cuboctahedral cages. Notably, along with the proposed interpretation, PALS results overestimate the sizes of all cages in DUT-67-Zr, while in DUT-67-Hf the size of the octahedral cage measured by PALS (0.90 nm) is almost the same as that predicted by PXRD. Moreover, PALS results indicate that the average size of cuboctahedral cages (1.50 nm) is very close to the larger cuboctahedral cages obtained from the crystal structure (1.48 nm) in DUT-67-Hf. The disparity in pore size estimation can be attributed to the surface morphology differences observed between DUT-67-Zr and DUT-67-Hf crystals. SEM images depicted the presence of distinct bumps forming shell-like layers on the surfaces of DUT-67-Zr crystals (Figure S7a). The formation of such shell layers in DUT-67-Zr may indicate secondary nucleation and crystallization. Conversely, the surfaces of the DUT-67-Hf crystals appeared significantly smoother (Figure S7b). These rough regions on DUT-67-Zr crystals likely contribute to the discrepancy in pore size estimation, potentially affecting the diffusion and behavior of Ps atoms within the material. Specifically, Ps is able to reach and annihilate in the pores in such shell layers on DUT-67-Zr, which might be of larger sizes than those in the core of the crystals (as the shell layers seem to be bright and less dense; Figure S7a). In this case, Ps can travel between the inner and shell porosity (Figure 3a), resulting in averaging the overall lifetimes in octahedral cages as well as in middle and large cuboctahedral cages to larger values corresponding to 0.99 and 1.8 nm, respectively. Additionally, these rough regions may bridge the particles, allowing the Ps atoms to travel between adjacent crystals and, again, averaging the Ps lifetimes (and hence pore sizes) to larger values. This behavior is absent in DUT-67-Hf thus Ps is only annihilating within the inner porosity (Figure 3b), or in interparticle spaces (as discussed below). Furthermore, the difference in the PALS data can be explained by the minor differences in the synthesis procedures (ZrOCl_2_x8H_2_O for DUT-67-Zr and HfCl_4_ for DUT-67-Hf) as well as small differences in the sample handling (washing procedure, desolvation) etc.

In both samples, a Ps lifetime (τ_5_ = 75 ns) equivalent to mesopores of size 6.0 nm is detected, which is missing in the crystal structure of the DUT-67 framework. The origin of this component can be directly attributed to Ps annihilation in widely spaced 6.0 nm crystal defects forming mesopores; especially its intensity is notable (8% in DUT-67-Zr and 20% in DUT-67-Hf in Figure 5b). This simplified interpretation closely aligns with the initial observation in IRMOF-1, where a resolved lifetime of 80 ns with an intensity ranging from 10% to 28% was reported.^55^ However, subsequent investigations on IRMOF-1 have demonstrated that this component originates from Ps diffusion from inner (framework) porosity to *interparticle spaces*.^56^ It has been described that Ps atoms in MOFs, having pores in the range of 1.3–1.5 nm, exist in a delocalized Bloch state with unprecedented mobility, allowing them to leave the particles. Similarly, in this context, τ_5_ is considered to originate from Ps trapping in *interparticle spaces* within DUT-67. Worth noting, the interparticle spaces between ∼0.4 μm average crystal sizes (Figure S7c and d from SEM) of DUT-67 series is expected to be larger than 6.0 nm and hence, the longest-lived lifetime, τ_5_, should be much closer to Ps lifetime in vacuum, 142 ns, not 75 ns. There are two possible explanations for such an underestimation of τ_5_. According to the results provided by Dutta and co-workers,^56^ Ps atoms in interparticle spaces in Zn_4_O coordinated polymer of ∼300 μm particle size (∼750 times the average particle size in DUT-67) have 0.1–0.3 eV energy boost. This energy allows Ps atoms to collide with approximately 100 particles. Therefore, the Ps atoms have a high probability of reentering the pores in the framework, averaging the Ps lifetime in a vacuum (142 ns) and in the framework (13 ns) to only 80 ns. Such an energy boost is expected to be even higher (due to less Ps thermalization) in DUT-67 of 0.4 μm average crystal size, allowing the Ps atom to visit more particles and resulting in an average lifetime of 75 ns. Another possibility that may also exist is that the longest-lived component overlaps with the background of random coincidences, presenting a difficulty in extracting it properly.^44^

To this end, the resolved Ps lifetimes originated from (i) octahedral cages (τ_3_), (ii) middle and large cuboctahedral cages (τ_4_), and (iii) Ps migration to interparticle spaces (τ_5_). This can be visualized in Figure 4.

In the *hydrated samples*, we first conducted PALS measurements for a duration of approximately 3 days at a certain RH value, namely, 9%, to explore the impact of humidity on porosity over time. Figure S2 illustrates an example of DUT-67-Zr at 9% RH, showing minimal changes in lifetimes and intensities during this period, which confirms the structural stability of DUT-67-Zr in the presence of humidity. In this stability test spanning 3 days, where we allowed 12 h for humidity levels to reach equilibrium, we conducted PALS measurements in slices of 5 h each. Subsequently, we compared the results of these individual slices collected over the three-day period with their summation. Additionally, we compared them with the results from a 3 h measurement (comprising 2 × 10^6^ events) to ascertain whether collecting higher statistics (in the 5 h slices or in the summation) would unveil any discernible differences. However, all results are comparable (see Figure S2 and Figure 5a), indicating that 2 × 10^6^ events are enough to resolve PALS spectra in DUT-67 MOFs, 12 h are sufficient to reach equilibrium, and no structural changes are observed over time. Therefore, we decided to expose the samples to humidity for 12 h, then proceeded to perform PALS measurements within 3 h. The 3 h PALS measurement approach is crucial to mitigate potential instabilities in the electronics during extended measurements.

The evolution of Ps lifetimes, τ_Ps_,(pore diameters) and intensities, I_Ps_, of DUT-67-Zr and -Hf with relative humidity from 0 (vacuum) to 100% is demonstrated in Figure 5a and b. For clarity, the discussions in the following section will focus on each pore group separately.

#### Interparticle Spaces

##### DUT-67-Zr

Water is expected to primarily adsorb in octahedral and cuboctahedral cages, and no change in the size of interparticle spaces is expected at low levels of RH until RH reaches 100%. Therefore, similar to the discussion in the literature,^44^ the longest-lived lifetime component in interparticle spaces was fixed to its dry value, 75 ns (interparticle spaces in Figure 5.a). This fixation reduces the uncertainties in other pore-related parameters with a low influence on their behavior or values.

The *I*_Ps_ (Figure 5.b) for interparticle voids is likewise predicted to remain constant until the inner porosity was filled. Surprisingly, it exhibits a rapid drop with increasing RH, reaching 0% at RH = 43%. Notably, this RH value coincides with the point at which Ps annihilation in the cuboctahedral cages ceases. These observations lead to the hypothesis that all Ps atoms that undergo annihilation in interparticle gaps are not formed there; rather, they originate inside and migrate out of the inner microporosity. The justification that Ps atoms annihilating in the interparticle spaces are mainly formed inside the crystals is that in DUT-67 (and in MOFs generally), there are no rigid pore walls to stop the positrons and allow them to interact with ionized electrons in order to form Ps. Instead, implanted positrons enter the particles via the spaces between the chains in the linkers and the Ps is expected to be formed inside the particles (Figure 5c, top). When the microporosity is filled or blocked, the Ps atoms can no longer reach the interparticle spaces. Therefore, the disappearance of the Ps lifetime component does not imply that the interparticle spaces have been filled but rather suggests that the Ps flow from inner porosity is hindered upon filling.

##### DUT-67-Hf

Here also, the ∼75 ns lifetime of the longest-lived component in the dry sample was resolved in interparticle spaces. Following the discussion about DUT-67-Zr, it has been fixed in DUT-67-Hf as well.

**Figure 6:**
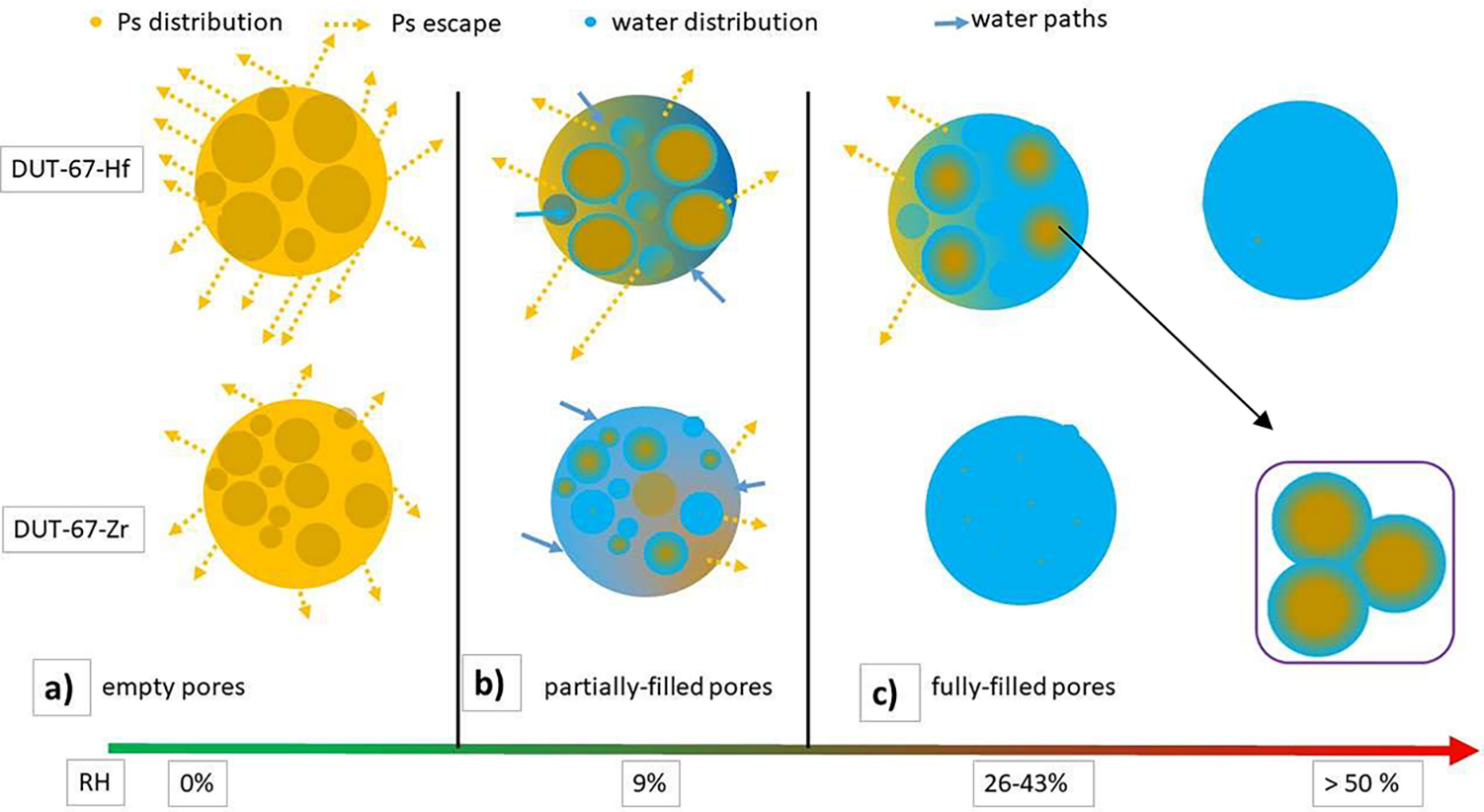
Evolution of pore filling by water (blue circles) after humidity exposure in DUT-67-Zr and -Hf having different routes (blue arrows) to water intrusion and the possible Ps distribution (yellow circles) and Ps escape (yellow arrows) starting from the (a) dry state and then (b) partial and (c) full filling of the pores. The circles in the box show the wetting of the inner surface of pores in DUT-67-Hf at RH = 43% and its influence on Ps distribution. The distributions shown in the figure are qualitatively, only.

The *I*_Ps_ within the interparticle spaces within DUT-67-Hf consistently exhibit higher values than their counterparts in DUT-67-Zr in both dry and hydrated states, extending up to RH = 34.5%. In the dry state, as discussed earlier, a meticulous examination of SEM results in Figure S7a and b unveils a notable distinction: DUT-67-Zr boasts a degree of surface roughness compared to DUT-67-Hf, yielding an augmented number of contact points between particles in DUT-67-Zr (Figure 3a). These contact points could allow the majority of Ps atoms to travel directly between adjacent particles, instead of occupying interparticle spaces and annihilate there. However, in the case of DUT-67-Hf where the roughness is absent, a high fraction of Ps atoms will annihilate in the interparticle spaces (Figure 3.b). This complex interplay between surface roughness and Ps behavior offers a possible explanation for the observed differences in *I*_Ps_. This behavior in interparticle spaces, i.e., *I*_Ps-Hf_ > *I*_Ps-Zr_, continues in the hydrated states until RH < 43%. Only at RH > 50%, water molecules are able to close the routes to the outer surface of the crystal, preventing the Ps from escaping, and *I*_Ps_ vanishes. The orange arrows in Figure 6 depict Ps escape to interparticle spaces during the pore filling process.

#### Cuboctahedral Cages

##### DUT-67-Zr

The drop in the τ_Ps_ value observed in the cuboctahedral cages (Figure 5a), from 17.5 ns at 0% RH to 10.4 ns at 9–26% RH, can be attributed to partial pore filling (Figure 6b). Additionally, a drop in τ_Ps_ due to changes in the electronic nature inside the pore due to adsorbed water, which alters the *pick-off* annihilation rate (further details in section S1), is anticipated. OH groups on the inner surface are known to reduce the Ps lifetime, thus contributing to this effect.^57^ The τ_Ps_ slightly increases in the RH range of 34.5%–43%. Since the τ_Ps_ is proportional to the pore size, an increase in τ_Ps_ would imply an expansion in pore size, which is physically not allowed in these MOFs. Building on the observations for the dry sample, τ_Ps_ in both middle and large cuboctahedral cages, which are close in size, is effectively averaged into a single value (cuboctahedral cages in Figure 5a). Beyond RH > 26%, the contribution of the middle cuboctahedral cages appears to diminish entirely, while the pure contribution of the larger cuboctahedral cages becomes more prominent. This shift in pore contributions results in a slight increase in τ_Ps_. Importantly, no discernible τ_Ps_ was resolved within the micropore range of cuboctahedral cages at RH > 43%, indicating complete pore filling (Figure 6c).

The I_Ps_ within the cuboctahedral cages (Figure 5b) decreases steadily with an increasing RH up to 26%, showing fewer empty pores due to humidity exposure (Figure 6b). The behavior shifts at RH > 26%, where the intensity stops decreasing at RH = 34.5%. This change might suggest the full filling of the middle cuboctahedral cages, with larger cuboctahedral cages coming into play. At RH = 43%, the *I*_Ps_ experiences a sudden drop to approximately 0% (explaining the large error bar of τ_Ps_ at RH = 43%), indicating the total filling of all cuboctahedral cages (Figure 6c). Further discussion on this is presented in the comparison with DUT-67-Hf below.

##### DUT-67-Hf

Interpreting τ_Ps_ results in cuboctahedral cages of DUT-67-Hf is challenging due to their distinct behavior with the Zr-containing sample. Notably, τ_Ps_ increases earlier in DUT-67-Hf (RH = 9%) than in DUT-67-Zr (RH > 26%). One plausible explanation for this early increase can be outlined as follows: Since middle and large cuboctahedral cage lifetimes are combined into a single component, this early increase at RH = 9% might stem from an early filling of the middle cuboctahedral cages in DUT-67-Hf. This early filling of the middle cuboctahedral cages will shift the average τ_Ps_ and pore size upward. This hypothesis is substantiated by the water isotherm data presented in Figure 2h, which clearly illustrates the earlier filling of middle cuboctahedral cages in DUT-67-Hf compared to DUT-67-Zr. This observation strongly implies an improved hydrophilic characteristic within the middle cuboctahedral cages of DUT-67-Hf, which may be explained by the minor differences in the synthesis procedures of the MOFs, such as metal precursors, and minor differences in the sample handling. However, the specific factors leading to differences in the hydrophilicity of the middle cuboctahedral cages between DUT-67-Zr and DUT-67-Hf remain somewhat enigmatic. Recently Lotsch and co-authors highlighted the reproducibility and complexity issues of Zr-MOF synthesis in the interlaboratory study pointing out that such discrepancies presumably arise from small changes in the synthesis environment that may not be easily noticed.^58^

The filling process of cuboctahedral cages proceeds until RH = 26%, after which the filling of even the larger cuboctahedral cages becomes significant, causing an overall decrease in average τ_Ps_ until the complete filling of all cuboctahedral cages at RH ≈ 50%. Besides the dissimilar behavior of τ_Ps_ between RH = 0–9% in Hf as compared to Zr in the same range, the disappearance of τ_Ps_ in cuboctahedral cages is shifted to RH = 50% (*vs* 43% in Zr). Although the steps in the isotherm are at the same *p*/*p*_0_, the difference may originate from the minor differences in the sample handling or from cluster formation, as discussed in Mechanism of Water Adsorption in DUT-67 below.

The I_Ps_ in DUT-67-Hf follows a similar trend compared with that of DUT-67-Zr until reaching 34.5% RH, showing a decline with increasing humidity. However, in this humidity range, *I*_Ps-Hf_ < *I*_Ps-Zr_, which can be explained from Figure 6, and it follows the discussion of *I*_Ps_ in interparticle spaces mentioned earlier. *I*_Ps-Hf_ < *I*_Ps-Zr_ in cuboctahedral cages because *I*_Ps-Hf_ > *I*_Ps-Zr_ in interparticle spaces as more Ps atoms are escaping from the cuboctahedral cages to interparticle spaces in DUT-67-Hf. Consequently, lower numbers (intensity) of Ps will annihilate inside the cuboctahedral pores in DUT-67-Hf compared to DUT-67-Zr. At RH = 43%, I_Ps_ in Hf grows 60% higher than its value at RH = 34.5%. This change is also associated with the change in I_Ps_ in interparticle spaces, as it almost diminishes at RH = 43%. Probably, the connecting path between interparticle spaces and cuboctahedral cages ceases to exist at this RH value. Due to the interactions between the water molecules and the metal cluster as well as the organic linkers, water molecules gradually bind to the inner surface of the pores and then start to occupy the small pores first. With this scenario, the other access to interparticle spaces from cuboctahedral cages, which were available at low RH values, is blocked at RH = 43% while some spaces of the inner part of cuboctahedral cages are still empty. In this case, the interparticle spaces will lose the majority of its Ps coming from cuboctahedral cages while the latter still have some empty spaces to host some Ps. This is better visualized in the section in the lower right-hand side of Figure 6. Complete filling of the cuboctahedral cages is achieved starting from RH = 50% as *I*_Ps_ is almost zero at this RH value, and it disappears at higher RH values.

#### Octahedral Cages

##### DUT-67-Zr

τ_Ps_ in octahedral cages in Figure 5a declines from 4.5 ns at 0% RH (under vacuum) to 1.5 ns at 100% RH, indicating progressive moisture adsorption. Notably, the τ_Ps_ at 100% RH and room temperature (1.5 ns) is obviously shorter than that of bulk water (1.87 ns^59^). The 1.87 ns lifetime of Ps in bulk water originates from Ps bubble formation in water^60^ due to the pressure exerted by Ps on the surrounding liquid causing a repulsion in the liquid molecules.^61^ Therefore, the shorter τ_Ps_ at 100% RH can be attributed to a suppressed mobility of water molecules to form liquid water due to their interaction with the skeleton. This assumption is consistent with previous discussions,^26^ where NPD pattern showed that the first D_2_O molecule is located at the μ_3_-O atom of the Zr-cluster (Figure 5c, bottom), while the second is located within the perimeter of the triangular octahedral cages (Figure 5c, middle). Adsorption on the first site is believed to be driven by the formation of O–D···O hydrogen bonds with the μ_3_-O(H) atoms of the Zr-cluster.^26^ This suggests that water molecules prefer to bind with these specific atoms rather than connect with one another. Therefore, the 1.5 ns lifetime measured in this range may indicate the physical size of the water-free voids as there is no indication of liquid water formation.

The *I*_Ps_ observed in the octahedral cages (Figure 5b) increases from 8% at 0% RH to 18% at 9% RH. At 0% RH, the structure is fully interconnected, and there are no obstacles to Ps motion. Consequently, Ps formed in the octahedral cages tends to migrate to larger pores, such as cuboctahedral cages and interparticle spaces, wherein the zero energy of Ps is lower than that in the octahedral cages. This results in a decrease in *I*_Ps_ in the octahedral cages at 0% RH. However, as the humidity increases to 9% RH, water molecules begin to accumulate in the octahedral cages, leading to a blockage that reduces the probability of Ps migration to larger pores and enhances the confinement of Ps in the octahedral cages. From 9% to 26% RH, there is a minor change in the I_Ps_ in the octahedral cages, indicating a slow filling of the octahedral cage window by water. Interestingly, there is a sharp decrease in I_Ps_ from 26% RH to 35% RH, suggesting that the Ps component designated as annihilation in the octahedral cages may also include a contribution from free volumes in the chains between the linkers. Therefore, it may be reasonable to conclude that the filling of the octahedral cages occurs at 26% RH, after which the detected I_Ps_ reflects free volumes between linkers. It should be noted here that starting from RH = 43%, all other Ps-related components in cuboctahedral cages and interparticle spaces disappear, as discussed previously, and only Ps annihilation in octahedral cages remain. However, the I_Ps_ in such single channels (octahedral cages) is insignificant. For instance, I_Ps_ = 5–2% between RH = 43 and 100%. This means that a tiny fraction of the injected positrons has been converted to Ps. In other words, the Ps formation probability has been significantly suppressed upon humidity exposure at RH > 43%. This could mean that either the majority of water-free spaces in the chains of the linkers have sizes smaller than the threshold free volume size required to form Ps (0.194 nm^62^) or Ps is oxidized by the hydroxyl radicals (• OH)^59^ between the chains. Ps oxidation means the dissociation of Ps atoms to e^+^, of a lifetime in the order to τ_2_ (∼500 ps), and e^–^.

##### DUT-67-Hf

Similar variations of τ_Ps_ and *I*_Ps_ in DUT-67-Zr are noticed for Hf metal ions. Notably, at 0% RH, the τ_Ps_ (indicative of pore size) is shorter in the case of Hf. This observation can be attributed to the absence of shell layers in DUT-67-Hf, as discussed earlier. These layers typically contribute to slightly larger pore sizes within the region of octahedral cages, leading to a slightly increased τ_Ps_ in the octahedral pores of DUT-67-Zr compared with those in DUT-67-Hf. Over the course of the humidity exposure, one can see that the τ_Ps_ values are identical for the Zr and Hf counterparts; hence, the discussion about Zr should be valid here as well. This indicates that the interactions between water molecules and the two DUT-67 hosts are equivalent, regardless of the metal ions. In this case, possibly the adsorption is dominated by physical interactions.

Since *I*_Ps_ magnitudes reflect the filling of pores (in terms of concentration) and the mutual connectivity between pores, their variation is determined by these two factors. Similar to Zr, the transition of *I*_Ps_ between 0% and 9% RH in the Hf case is based on the blockage of paths to larger pores and partial confinement of Ps in the octahedral cages. Though, when RH = 9–26%, *I*_Ps-Hf_ < *I*_Ps-Zr_. This can be understood, similar to the discussion about cuboctahedral cages, if we recall Figure 3b and *I*_Ps_ in interparticle spaces (Figure 5b). One possible explanation for the decreased *I*_Ps_ in the octahedral cages of DUT-67-Hf involves the increased migration of Ps from these pores to the interparticle spaces, where they eventually undergo annihilation. This enhanced migration process is facilitated by the absence of shell layers in DUT-67-Hf, which contrasts with the configuration in DUT-67-Zr. In DUT-67-Zr, these shell layers enable some Ps to traverse between adjacent crystals and ultimately reach the octahedral pores in neighboring particles, increasing I_Ps_ in octahedral cages of DUT-67-Zr as compared to DUT-67-Hf. An abrupt drop in *I*_Ps_ is observed at RH > 34.5% suggesting a filling of octahedral cages while the contribution from free volumes between linkers is retained at higher RH as explained for the Zr example. This indicates that the filling of octahedral cages in Hf shifts to higher RH value (∼35%) compared to Zr (26%). The origin of such a delayed filling of the octahedral cages in Hf counterpart is, like cuboctahedral cages, still ambiguous and may be explained by minor differences in sample handling.

### Mechanism of Water Adsorption in DUT-67

Finally, in order to provide a complete mechanism of pore filling in DUT-67 MOFs, it is important to compare PALS data with NPD and water adsorption isotherms. In our previous work^26^ on DUT-67-Zr, the NPD results indicate the mechanism of pore filling. Three distinct adsorption steps during the course of water adsorption are resolved: The sample with loading 1 shows only one preferable adsorption site close to the Zr-cluster and another one in the octahedral cages; the sample with loading 2 indicates the nearly complete filling of octahedral cages and start filling of middle cuboctahedral cages; and loading 3 indicates the filling of large cuboctahedral cages. Similar results are also expected in DUT-67-Hf (because of the similar isotherms). This is in perfect agreement with the current findings by PALS, which also show that water adsorption takes place progressively, starting with the filling of the smallest pores (octahedral cages), followed by the middle cuboctahedral and finally the larger cuboctahedral pores. Additionally, comparing the steps of complete filling of each pore group as driven from PALS with those obtained from water adsorption isotherms at room temperature in Figure 2h may reveal new insights. Note that we mean by complete filling in water isotherms the flipping point between two adjacent steps. Table 2 summarizes these steps.

**Table 2:** Steps of DUT-67-Zr and -Hf Pore Filling by Water As Obtained from Water Adsorption Isotherms and an In Situ Humidity Experiment During PALS Measurements

	Octahedral cages	Middle cuboctahedral cages	Large cuboctahedral cages
DUT-67-Zr			
RH-PALS	0.26	0.34	0.43
*p*/*p*_0_ – water adsorption	0.30	0.37	0.45
DUT-67-Hf			
RH-PALS	0.35	0.09–0.50	0.50
*p*/*p*_0_ – water adsorption	0.25	0.35	0.45

As observed in Table 2:

(i) The concordance is evident in DUT-67-Zr, where the differences between the humidity steps observed in PALS experiments and the corresponding relative pressure values from the isotherms are minimal during the filling process. However, DUT-67-Hf shows mismatch at high loadings.
(ii) In DUT-67-Hf, the RH values identified from PALS for the filling of each pore group are broader than their respective relative pressure values in the isotherm experiment.
(iii) PALS results suggest that the filling of pores in DUT-67-Hf is shifted toward higher RH values compared to DUT-67-Zr. This shift may be due to the minor differences in pore surface properties, such as hydrophilicity, possibly influenced by sample handling.

To elucidate the observations mentioned above, it should be noted that the steps of complete filling from the PALS results are based on the transitions in τ_Ps_ or *I*_Ps_. In this regard, if the pore filling process proceeds via layer-by-layer mechanism, one should anticipate observing a gradual decrease in τ_Ps_ and I_Ps_^58^ in cuboctahedral and octahedral cages as the free volume fills. This decline eventually reached a minimum value corresponding to Ps annihilation in water. However, Ps parameters within these cages are resolved up to a specific humidity level, after which they abruptly vanish. The vanishing of τ_Ps_ and I_Ps_ in cuboctahedral and octahedral cages (at RH = 35–50%, see Figure 5) is not associated with resolving τ_Ps_ in water (1.87 ns). Instead, the minimum resolvable τ_Ps_ lies between 3.5 and 2.5 ns (Figure 5a, lower panel). This behavior implies that Ps annihilates in water-free voids at low RH levels, whereas at higher RH values, it annihilates in small and confined cavities within the pores, leading to τ_Ps_ in the range of 2.5 to 3.5 ns. A more reasonable scenario describing the process involves water molecules adhering to the pore surface due to their preference to bind with metal ions, as discussed in the results of octahedral cages, forming water clusters at low RH levels. These clusters are expected to expand in size and quantity with increasing RH, accumulating to form small air gaps (cavities) in the pores. These cavities are expected to persist even under high water loadings, as explained earlier in the results of octahedral cages. The manifestation of cluster formation during water adsorption is evident in the isotherm presented in Figure 2h, which is characterized by distinct steep steps. Moreover, the formation of clusters in CAU-10-H and CAU-10-CH_3_ framework was experimentally proved by van der Veen et al.^63^ Authors point out that minor changes in the crystal structure strongly influence adsorption properties, assigned to the formation of energetically far more favorable water clusters in CAU-10-H.

Now back to point *i* above, the mismatch noticed between PALS and water isotherms resulting in DUT-67-Zr and -Hf can be explained in terms of the clustering effect. In the spaces between clusters, Ps can continue to form and explore any unfilled areas. Ps formation will be significantly inhibited only when the clusters accumulate and saturate the entire pore surface, as indicated by the *I*_Ps_ values in Figure 5b for RH > 50%. Hence, the mismatch in the case of DUT-67-Hf implies that the transition from stable clusters to accumulated clusters with small cavities occurs at RH values higher than DUT-67-Zr. Consequently, the indications about complete filling from Ps data will be shifted to higher RH values as well, thereby explaining point *i*. This shifted transition from stable clusters to distorted ones in DUT-67-Hf may be based on its stronger hydrophilicity. The stronger hydrophilicity of the DUT-67-Hf sample is confirmed by the steep step in the water adsorption isotherm at *p*/*p*_0_ = 0.28, compared to the step at *p*/*p*_0_ = 0.33 in the DUT-67-Zr isotherm.

The broadness of the RH values identified from PALS for the filling of each pore group (point *ii*) and the shift of the filling of pores in DUT-67-Hf toward higher RH values compared to DUT-67-Zr (point *iii*) can also be interpreted from the clustering effect.^63^ By recalling the physical sizes of Ps (∼0.15 nm) and water molecules (0.275 nm), it is evident that Ps atoms can penetrate through smaller sizes than water. While the clusters remain stable, the spaces between adjacent clusters likely serve as pathways for Ps atoms, enabling them to access and occupy any empty spaces around the water clusters. However, these pathways are insufficient to accommodate the movement of free water molecules into any remaining empty spaces; instead, the water molecules tend to attach to existing clusters, causing them to grow in size. This scenario can elucidate point *ii*. At high relative pressure of water (and RH) the packing density of adsorbed water molecules in all pores reaches its maximum. Consequently, the clusters collapse and aggregate, filling all pores with small cavities in water. This process occurs at RH ≥ 50% in DUT-67-Hf. In contrast, the clustering effect is expected to be less pronounced in DUT-67-Zr, as evidenced by the agreement between the PALS and the water isotherm data. In this case, water molecules are more likely to form continuous islets throughout the entire pore space. These isles coalesce efficiently, filling all pores smoothly at RH values lower than those in DUT-67-Hf. As a result, Ps characteristics do not significantly deviate from those observed during water adsorption, explaining point *iii*. This highlights the sensitivity of Ps to the evolving nature of the water loading process in MOFs.

## Conclusions

This work offers valuable insights into the water adsorption process in environmentally benign DUT-67 systems, which hold promise for applications such as atmospheric water harvesting and adsorption-driven heat pumps. The mechanism of water uptake in chemically stable DUT-67 MOF variants, featuring Zr and Hf clusters as metal nodes, has been elucidated through a synergistic combination of PALS, PXRD, N_2_ adsorption, and water adsorption techniques. In situ humidity exposure during PALS measurements revealed a sequential filling process, starting with the octahedral cages, followed by the middle cuboctahedral cages and concluding with the large cuboctahedral cages. This observation aligns perfectly with the results obtained from the water adsorption and NPD experiments. Furthermore, the structural stability of DUT-67 samples, when loaded with water, was confirmed during a 3 day PALS run, demonstrating their suitability for practical applications. Moreover, the differences observed between PALS and water vapor physisorption measurements have revealed additional insights into the adsorption process. The higher humidity levels required for pore filling in DUT-67-Hf, as compared to DUT-67-Zr, can be attributed to differences in hydrophilicity of the samples, which cannot be explained by any of the applied characterization techniques and presumably originated from minor differences in the synthesis procedures and sample handling. PALS results suggest that the clustering effect in DUT-67-Hf appears to be more pronounced than that in DUT-67-Zr. In DUT-67-Hf, this clustering effect causes water molecules to adhere to the inner pore surfaces. Conversely, in DUT-67-Zr, water molecules aggregate into isles that primarily occupy the entire pore space. These isles in DUT-67-Zr merge to fill all of the pores without obstructing any empty spaces. In contrast, water clusters in DUT-67-Hf limit the filling of certain unfilled spaces, but Ps can still reach and detect these obstructed regions. Only at an RH of 50% in DUT-67-Hf is the saturation point reached, and all spaces become filled with water, having some small unfilled cavities. This intriguing behavior highlights the significance of considering Ps atoms as probing entities in pore characterization studies as they can provide distinct information not obtained solely from adsorption measurements. Remarkably, the present work shows how Ps intensity acts as an index of vapor adsorption in microporous systems.

## Supporting Information Available

Information on basics of positron annihilation process and PALS porosimetry, chemical composition of the used saturated salt solutions, records of humidity levels recorded during PALS measurements, an assessment of the structure stability of the MOF samples during humidity exposure as expected from PALS, results from N_2_ adsorption and TGA measurements, and selected SEM images showcasing the crystal morphologies and particle size distributions of DUT-67-Zr and -Hf samples (PDF)
