# Supplementary material for: Unravelling the Water Adsorption Mechanism in Hierarchical MOFs: Insights from In Situ Positron Annihilation Lifetime Studies
Source: ACS Appl Mater Interfaces. 2023 Oct 5;15(41):48264–76. doi: 10.1021/acsami.3c10974 (PMC10591278; doi:10.1021/acsami.3c10974)
Supplement: Supplementary file 1 — am3c10974_si_001.pdf [file am3c10974_si_001.pdf]

Supporting Information

Unravelling the water adsorption mechanism in hierarchical MOFs: insights from in situ positron annihilation lifetime studies

Ahmed G. Attallah[1,2,], Volodymyr Bon[3,*], Kartik Maity[3], Eric Hirschmann[2], Maik Butterling[2], Andreas Wagner[2], Stefan Kaskel[3,4]*

[1]Helmholtz-Zentrum Dresden-Rossendorf, Institute of Radiation Physics, 01328, Dresden, Germany

[2]Physics Department, Faculty of Science, Minia University, 61519, Minia, Egypt

[3]Chair of Inorganic Chemistry I, Technische Universitat Dresden, Bergstrasse 66, D-01062 Dresden, Germany.

[4]Fraunhofer Institute for Material and Beam Technology IWS, Winterbergstraße 28, D01277, Dresden, Germany

Corresponding authors' email addresses: a.elsherif@hydr.de (Ahmed G. Attallah);

volodymyr.bon@tu-dresden.de (Volodymyr Bon)

Unravelling the water adsorption mechanism in hierarchical MOFs: insights from in situ positron annihilation lifetime studies

Ahmed G. Attallah[1,2,], Volodymyr Bon[3,*], Kartik Maity[3], Eric Hirschmann[2], Maik Butterling[2], Andreas Wagner[2], Stefan Kaskel[3,4]*

[1]Helmholtz-Zentrum Dresden-Rossendorf, Institute of Radiation Physics, 01328, Dresden, Germany

[2]Physics Department, Faculty of Science, Minia University, 61519, Minia, Egypt

[3]Chair of Inorganic Chemistry I, Technische Universität Dresden, Bergstrasse 66, D-01062 Dresden, Germany.

[4]Fraunhofer Institute for Material and Beam Technology IWS, Winterbergstraße 28, D01277, Dresden, Germany

S.1. Positron annihilation lifetime spectroscopy

Energetic positrons (antiparticles of electrons) of ~540 keV endpoint energy (^{22}Na positron sources) diffuse in materials until a certain depth depending on the material's density and defect content. During diffusion, positrons lose their energy by inelastic collisions with atoms and in the end, they become thermalized particles. Thermalized positrons are attracted to and trapped in open-volume defects, voids in material's matrix, interparticle spaces, and open and closed pores (because of the missing repelling nuclei) [1]. Such a sensitivity to free volumes enables positron to probe all kinds of free volumes in materials. Positron annihilation is recognized by detecting

two 511 keV gamma-photons that are emitted when the wavefunctions of positrons and electrons overlap. Positron annihilation lifetime is a measure of the time elapsed since positron implantation until its annihilation with one electron. The elapsed time is governed by the probability of how often a positron meets an electron (electron density distribution) at the trapping site.

On one hand, free (unbound) positrons can annihilate with electrons from the bulk or from defects. Since this probability depends on the size of the defective site, the distinguishability between these traps (monovacancy, vacancy cluster) is based on the value of the positron lifetime. On the other hand, positrons can also bind with electrons in porous solids with free space > ~ 2 Å [2] and on inner pore walls while forming the so-called 'positronium (Ps)' atom before annihilation. Ps has two states that depend on the spin alignment of electron and positron; para-positronium (p-Ps) and ortho-positronium (o-Ps). p-Ps is a singlet state with antiparallel spins and it decays into two-511 keV photons with 0.125 ns lifetime in vacuum. While o-Ps is a triplet state with parallel spins and annihilates into three photons with an intrinsic lifetime of 142 ns in vacuum. The short-lived and self-annihilating p-Ps is weakly affected by the surrounding media [3] hence it cannot be used for measuring the size of free volumes (pores). It is common in the literature to fix τ_1 to 125 ps (theoretical lifetime of p-Ps) in order to minimize the uncertainties associated with the pore-related components. However, this approach may be flawed as τ_1 could also include contributions from the spaces between the chains in the organic linkers, which might depend on humidity level. Due to the limited timing resolution (240 ps), distinguishing between the very similar lifetimes of p-Ps and unbound positrons between the chains is not feasible. As a result, we made the decision to keep τ_1 as a free parameter in our analysis. It is worth noting that both τ_1 and τ_2 are independent of the pore structure and are therefore excluded from the discussion in the main text.

The sufficiently long-lived o-Ps is capable of approaching the pore wall many times before annihilation and its lifetime is significantly reduced depending on the size of the probed free volumes. This is because trapped o-Ps in free volumes annihilates by the 2-gamm mode when the positron involved in o-Ps finds an electron in the pore wall with an antiparallel spin. This process is known as *pick-off* annihilation [4]. The *pick-off* annihilation probability (reciprocal of o-Ps

lifetime) is therefore large for small free volumes. This means that this collisionally-reduced o-Ps lifetime provides the physical basis for probing free volumes by PALS [5]. The correlation between o-Ps lifetime and pore size has been first described in the Tao-Eldrup (TE) model [6,7], which is valid only for spherical micropores (R > 1 nm). Later, the TE-model had been extended to include larger pores of different pore shapes and at any temperature in the Rectangular TE (RTE) model [4] and in the Extended TE (ETE) model [8]. According to the TE model, the Ps atom is supposed to be trapped in holes of spherical shape surrounded by an infinite potential well. As mentioned above, the TE model is valid to probe hole radii < 1nm because of the huge difference between the ground state and the excited states which restricts the TE calculation to the Ps atom populating the ground state only. The relation between the measured o-Ps lifetime ($\tau_{o\text{-}Ps}$) and pore radius (R) [6,9] is expressed as;

$$\tau_{o-Ps} = 0.5\, ns\, [1 - \frac{R}{R+\delta} + \frac{1}{2\pi}\sin(\frac{2\pi R}{R+\delta})]^{-1} \qquad (1)$$

The 0.5 ns is the spin-averaged lifetime of the Ps, and the empirically determined δ) 1.66 Å describes the penetration of the Ps wave function into the hole "walls".

Table S1. Saturated salt solution and relative humidity values used for in situ PALS measurement.

Saturated salt solution	LiCl	CH_3COOK	$CaCl_2$	K_2CO_3	$Ca(NO_3)_2$	NH_4NO_3	NaCl	NH_4Cl
RH (%)	9	26	34.5	43	51	63.5	75	79

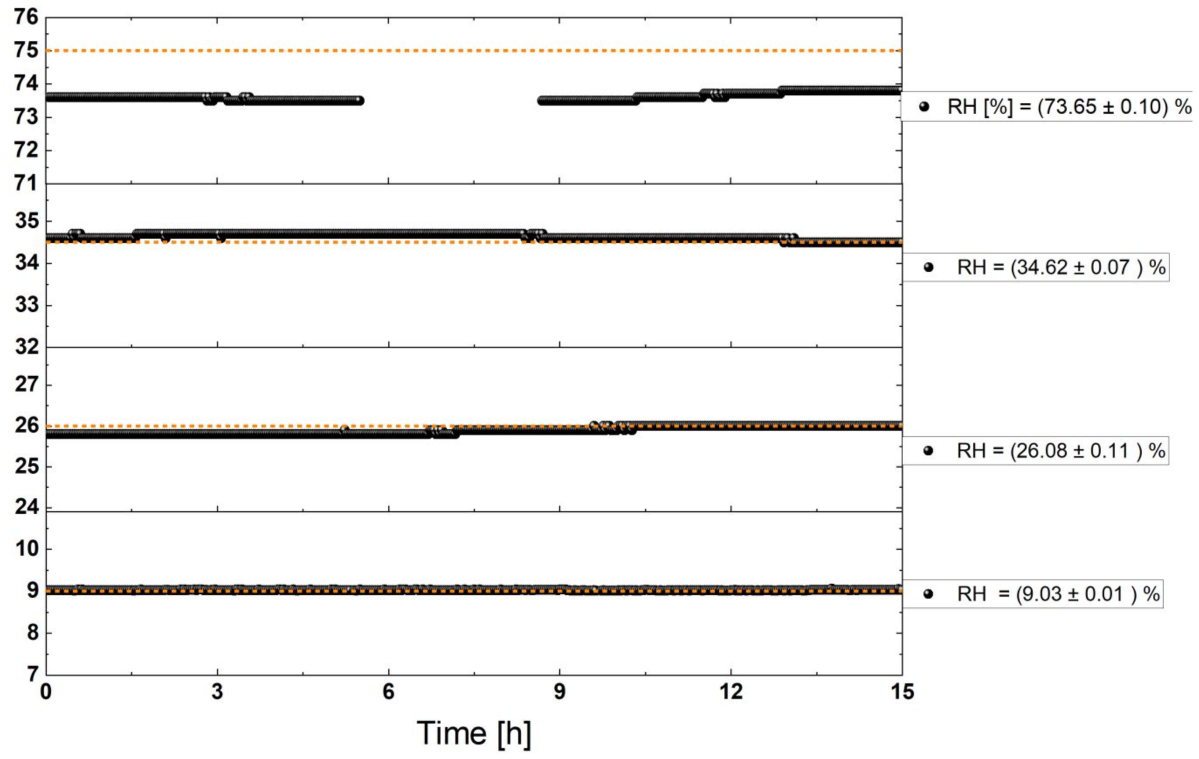

Figure S1. Examples of the relative humidity monitored by the humidity sensor during PALS measurements. Values right to the figure show the average RH. Target RH values are marked with the orange dotted lines.

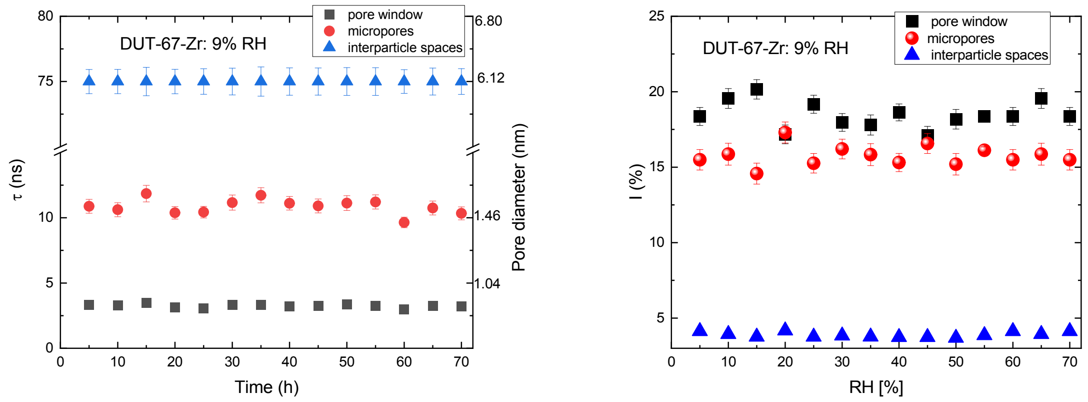

Figure S2. Variation of positronium lifetimes (left) and intensity (right) in pore window, micropores, and interparticle spaces of DUT-67-Zr MOF when in situ exposed to 9 % RH for ~ 3days. Pore diameters, by assuming spherical pore shapes, are given on the right axis.

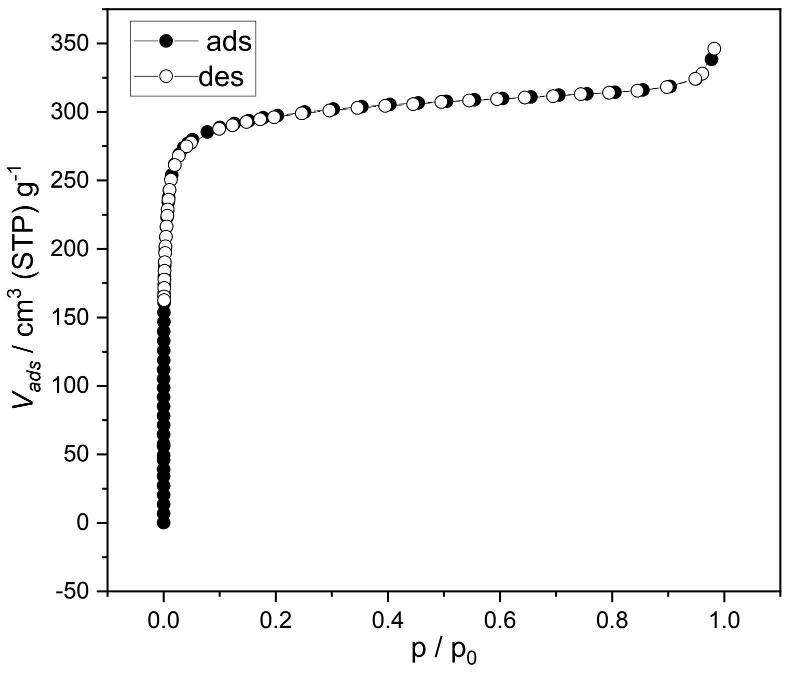

Figure S3. N_2 physisorption isotherm for desolvated DUT-67-Zr at 77 K: V_p = 0.57 cm^3g^{-1} at P/P_0 = 0.9; S_{BET} = 1143 m^2/g (Calculated from the nitrogen adsorption branch below P/P_0. = 0.3)

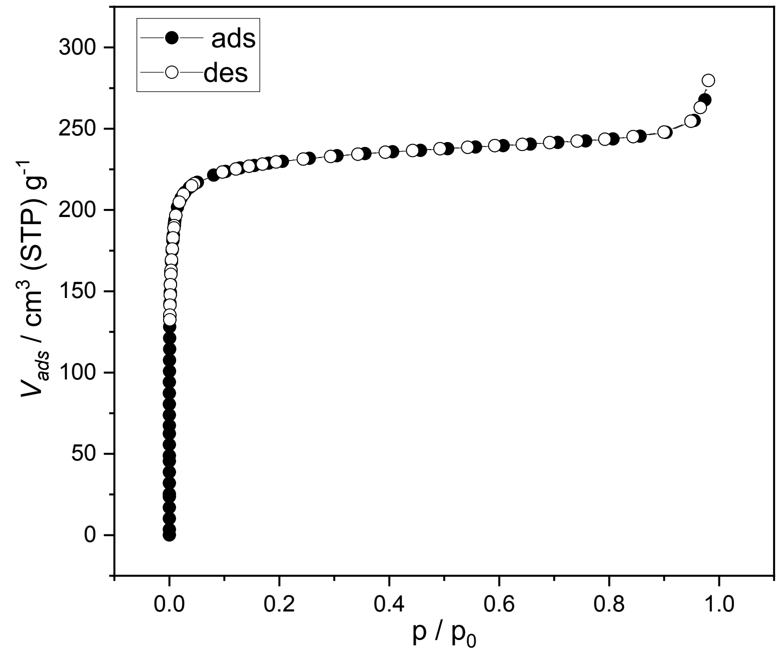

Figure S4. N_2 physisorption isotherm for desolvated DUT-67-Hf at 77 K: V_p = 0.43 cm^3g^{-1} at P/P_0 = 0.9; S_{BET} = 863 m^2/g (Calculated from the nitrogen adsorption branch below P/P_0. = 0.3)

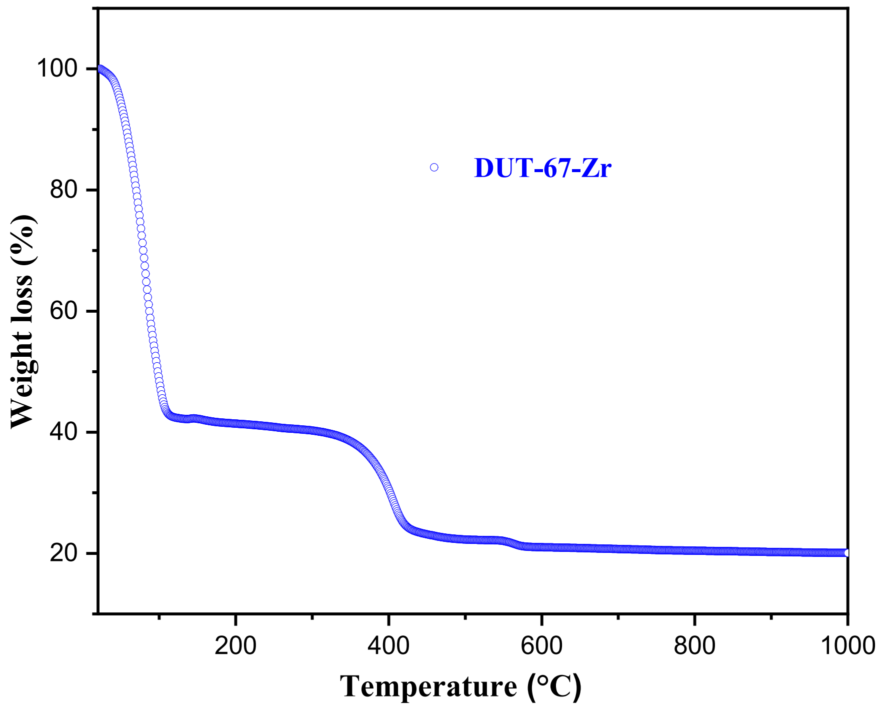

Figure S5. TG of as-synthesized DUT-67-Zr under the flow of synthetic air.

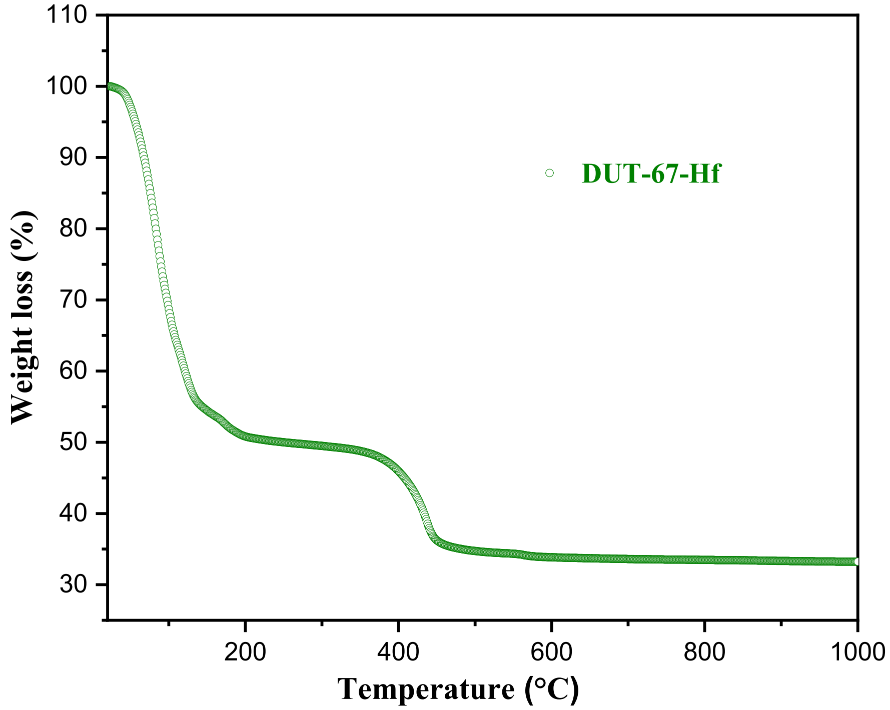

Figure S6. TG of as-synthesized DUT-67-Hf under the flow of synthetic air.

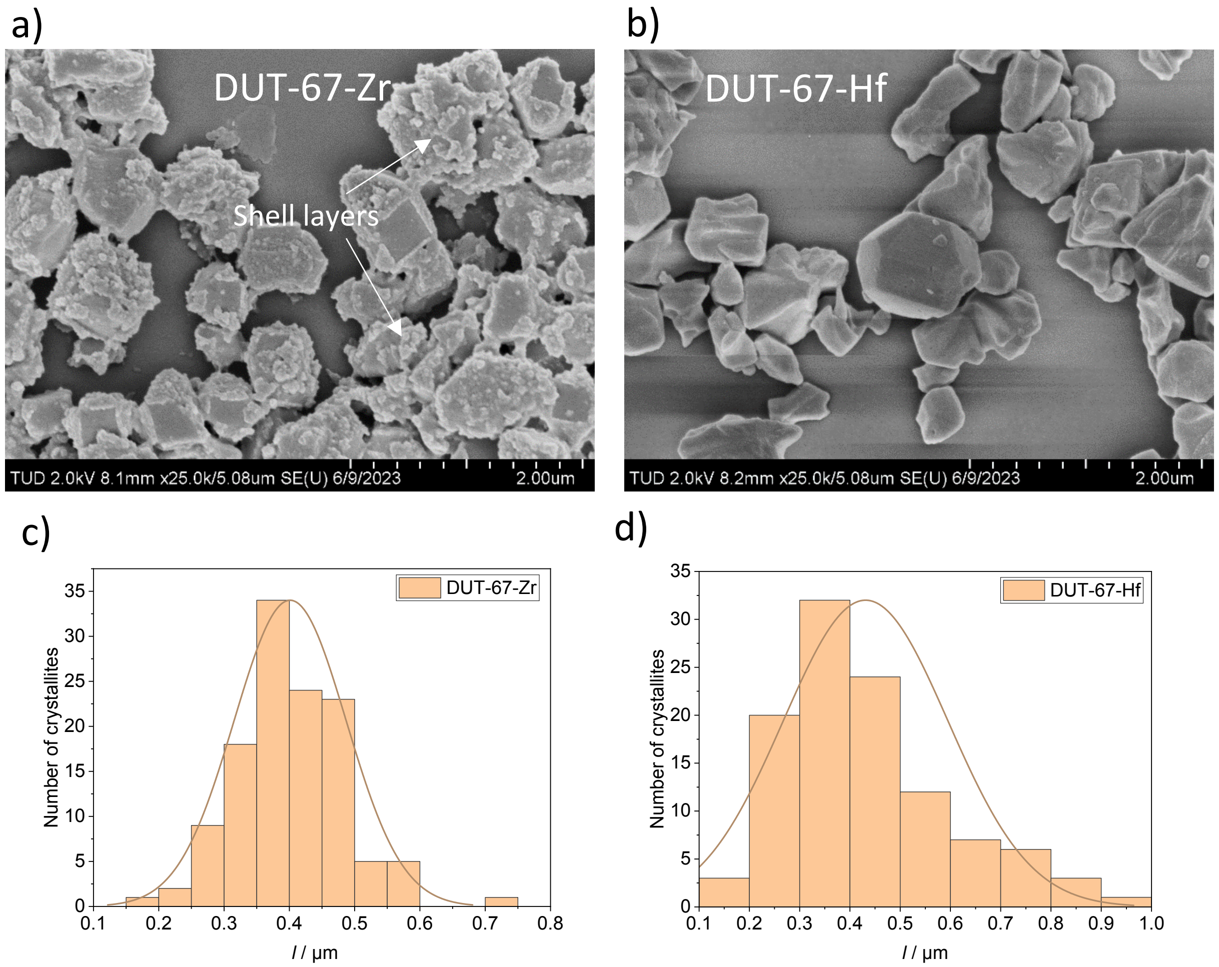

Figure S7. Selected SEM images for DUT-67-Zr (a) and DUT-67-Hf (b) showing the smoothness of their outer surfaces. Crystal size distribution for DUT-67-Zr (c) and DUT-67-Hf (d), calculated from SEM images.
